# Supplementary material for: Tumor Treating Fields Alter the Kinomic Landscape in Glioblastoma Revealing Therapeutic Vulnerabilities
Source: Cells. 2023 Aug 30;12(17):2171. doi: 10.3390/cells12172171 (PMC10486683; doi:10.3390/cells12172171)
Supplement: Supplementary file 1 [file cells-12-02171-s001.zip › Supplemental Figure S1.pdf]

Supplemental Figure S1: Differential Growth Patterns of Parental and TMZ Resistant Cells.

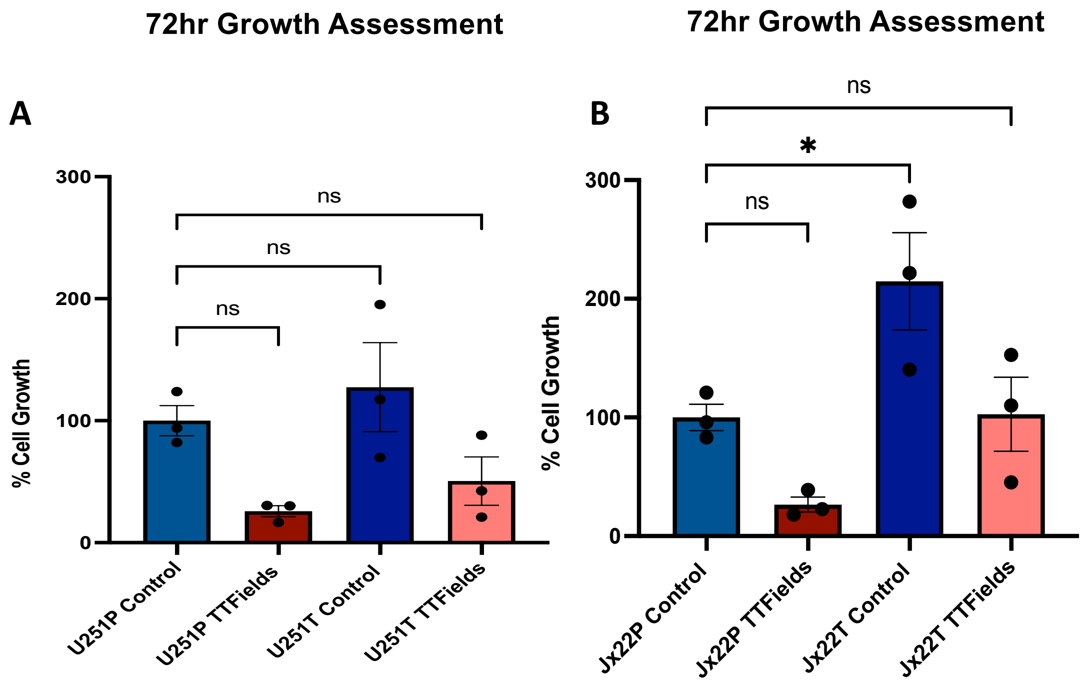

Supplementary Figure S2. Differential Growth Patterns of Parental and TMZ Resistant Cells. Data from the basal growth experiments (Figure 1) in the presence or absence of TTFields were re-analyzed for the U251 (A) and Jx22 (B) GBM models to determine differences in growth independent of TTFields. All experimental conditions were normalized to their respective control treated parental cells to determine growth trends. Data are displayed as means ± SEM (n=3 biological replicates performed in technical duplicates). ns=nonsignificant, * $P \leq 0.05$.
