# Supplementary material for: Tumor Treating Fields Alter the Kinomic Landscape in Glioblastoma Revealing Therapeutic Vulnerabilities
Source: Cells. 2023 Aug 30;12(17):2171. doi: 10.3390/cells12172171 (PMC10486683; doi:10.3390/cells12172171)
Supplement: Supplementary file 1 [file cells-12-02171-s001.zip › Supplemental Figure S2.pdf]

n=4 bio rep.

Supplemental Figure S2: CK2α Phosphorylation is Decreased Following Treatment with TTFields

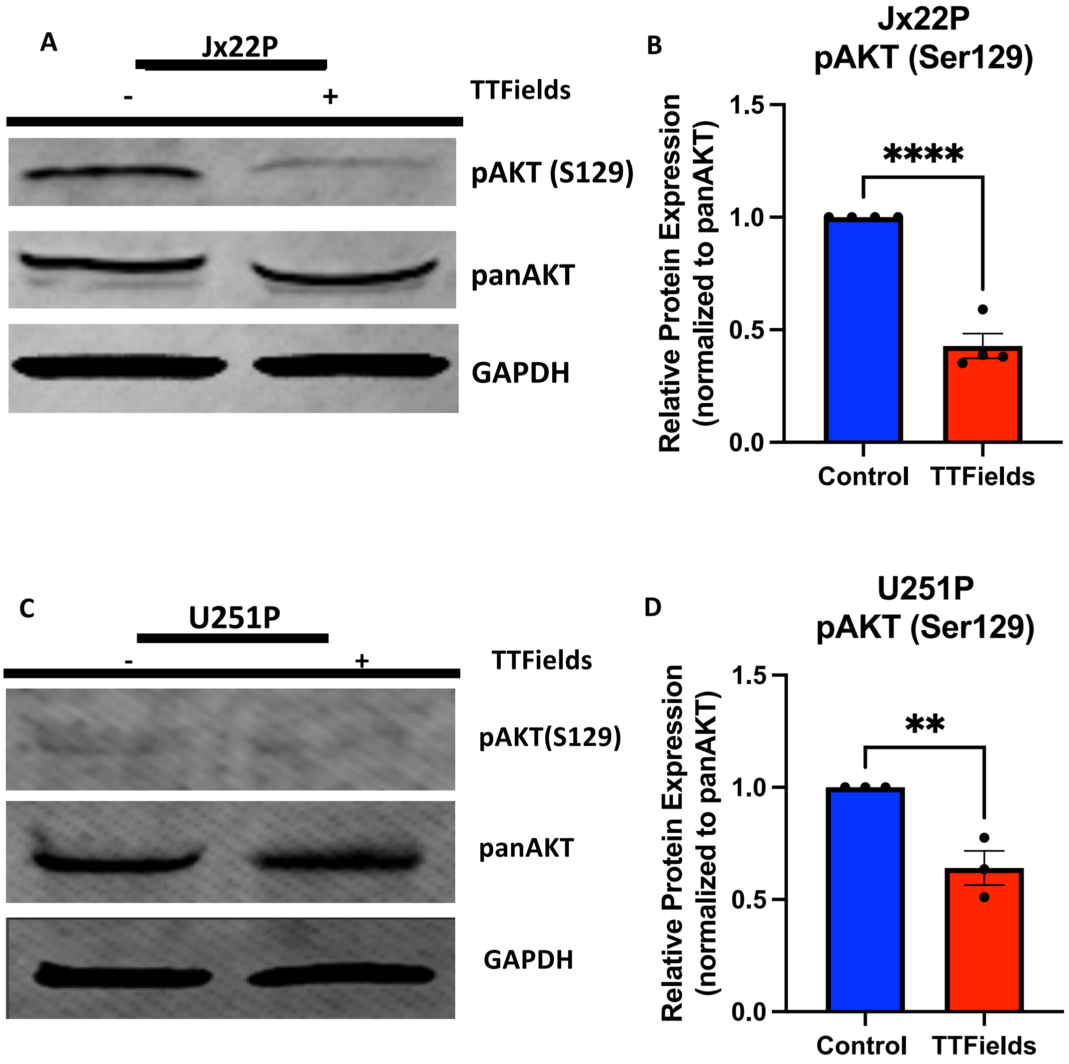

Supplementary Figure S2. CK2α Phosphorylation is Decreased Following Treatment with TTFields. Protein expression of pAKT (S129), panAKT, and GAPDH in Jx22P (A) and U251P (C) lysates harvested from control or 4-hour TTField treated cells. Densitometry values (n= 4 biological replicates for Jx22P and n=3 for U251P) for each protein was determined using ImageStudio Lite software. Quantification of densitometry values for pAKT(S129) in Jx22P (B) and U251P (D) TTField samples were normalized to control treated panAKT values. Data are displayed as means ± SEM. ** $p < 0.01$, **** $p < 0.0001$ with an unpaired *t*-test.
