# Supplementary material for: Tumor Treating Fields Alter the Kinomic Landscape in Glioblastoma Revealing Therapeutic Vulnerabilities
Source: Cells. 2023 Aug 30;12(17):2171. doi: 10.3390/cells12172171 (PMC10486683; doi:10.3390/cells12172171)
Supplement: Supplementary file 1 [file cells-12-02171-s001.zip › Supplemental Figure S3.pdf]

Supplemental Figure S3: Approximate IC50s for Crenolanib in Utilized GBM Models

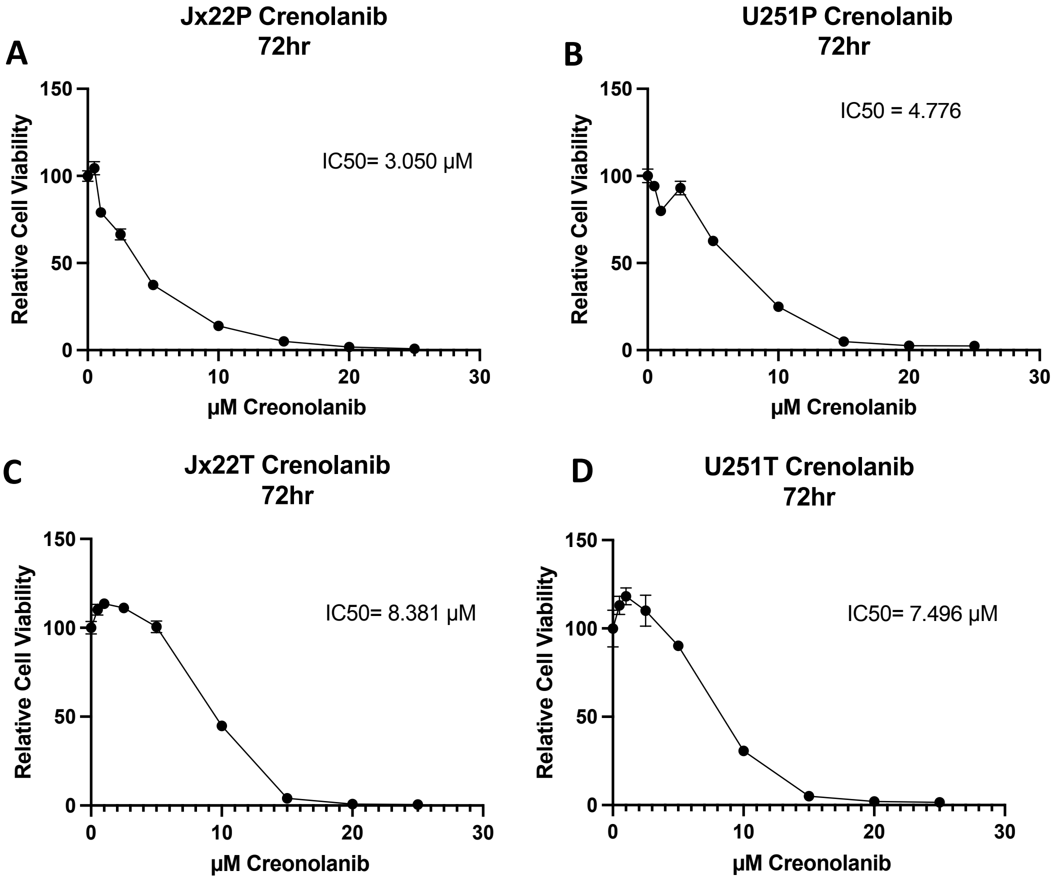

Supplementary Figure S2. Approximate IC50s for Crenolanib in Utilized GBM Models. TMZ sensitive GBM cells (A and B) and TMZ resistant (C and D) were treated with increasing concentrations of Crenolanib (0-25µM) for 72-hours and relative viability was determined using Cell Titer Glo. Graphs are representative of a single experiment, performed in technical triplicate. Data is normalized to DMSO control and individual concentrations are displayed as means ± SEM.
