# Supplementary material for: Tumor Treating Fields Alter the Kinomic Landscape in Glioblastoma Revealing Therapeutic Vulnerabilities
Source: Cells. 2023 Aug 30;12(17):2171. doi: 10.3390/cells12172171 (PMC10486683; doi:10.3390/cells12172171)
Supplement: Supplementary file 1 [file cells-12-02171-s001.zip › Supplemental Figure S4.pdf]

Supplemental Figure S4: Complete western blot images for supplemental figures.

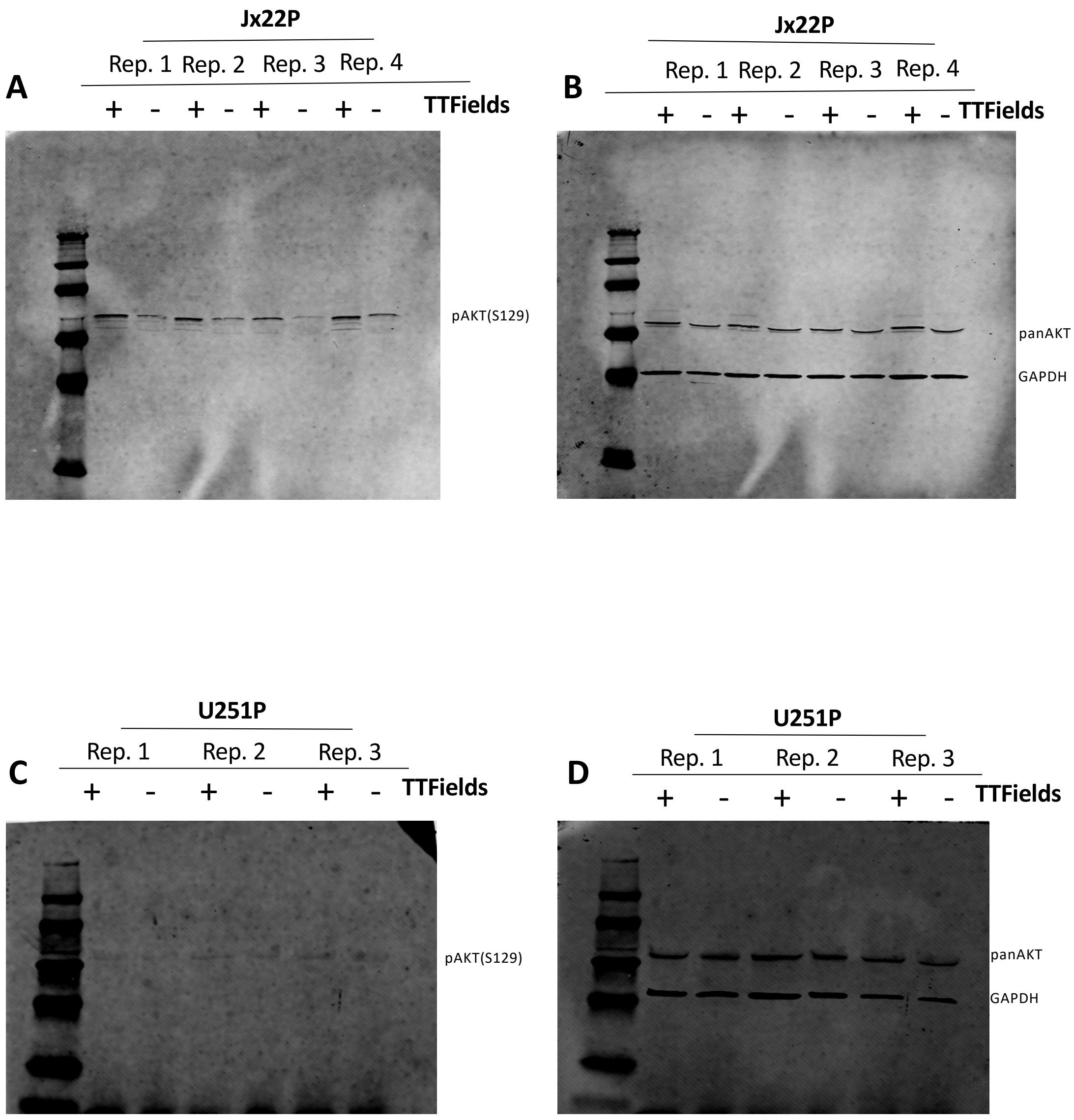

Supplementary Figure S4. Complete western blot images for supplemental figures. Full sized western blots for supplemental figure S2. All replicate samples for both Jx22P (A and B) and U251P (C and D) cells were run on the same blot and probed for pAKT(S129) first and then stripped and re-probed for panAKT and GAPDH together.
